# Evaluation of the theoretical optimal angle of the tibial tunnel in transtibial anatomic posterior cruciate ligament reconstruction by computed tomography

**DOI:** 10.1186/s12891-018-2348-4

**Published:** 2018-12-06

**Authors:** Xiaohui Zhang, Yuanjun Teng, Xinxin Yang, Rui Li, Chongwen Ma, Hong Wang, Hua Han, Bin Geng, Yayi Xia

**Affiliations:** 1 Department of Orthopaedics, Lanzhou University Second Hospital, Lanzhou University, No. 82 Cuiyingmen, Chengguan District, Lanzhou City, Gansu Province 730030 People’s Republic of China; 2 Orthopedics Key Laboratory of Gansu Province, Lanzhou University Second Hospital, Lanzhou University, No. 82 Cuiyingmen, Chengguan District, Lanzhou City, Gansu Province 730030 People’s Republic of China

**Keywords:** Posterior cruciate ligament, Reconstruction, Tunnel angle, Computed tomography

## Abstract

**Background:**

“Killer turn” effect is a critical explanation for the recurrent posterior laxity following transtibial posterior cruciate ligament (PCL) reconstruction, which affected by the angle of the tibial tunnel. Meanwhile, excessive tunnel angle would have an adverse impact on the healing of tendon to bone. The purpose was to evaluate the theoretical optimal angle of the tibial tunnel in transtibial anatomic PCL reconstruction.

**Methods:**

The measurements were performed on CT sagittal plane, including the thickness of cancellous bone (L1), the theoretical optimal angle of the tibial tunnel (TOA, which was measured between tibial plateau and the extension cord connecting the center of PCL insertion site with a point 5 mm superior from marrow cavity vertex), L2 - the distance from anterior tunnel aperture to anterior end of tibial plateau, L3 - the distance from anterior tunnel aperture to tibial tuberosity (lowest edge of patellar ligament attachment).

**Results:**

The value of TOA and L3 were 35.4 ± 7.9 ° and 26.8 ± 11.4 mm, respectively. L1 and L2 were higher in males than females (L1, *P =* 0.002*;* L2, *P =* 0.046). Regarding age, L1, TOA, L2 and L3 were higher in the 46–60 years group than 31–45 years group (*P* = 0.02, *P* = 0.001, *P* = 0.038, *P* = 0.032, respectively). With regard to height, L1 was lower in group I - < 1.66 m than group II - 1.66 to 1.75 m and group III - > 1.75 m (I v II, *P* = 0.015, I v III, *P* = 0.026). L2 was also lower in group I than group II and group III (I v II, *P* = 0.026, I v III, *P* = 0.006). TOA and L3 showed no significant differences among sex and height groups (*P > 0.05*).

**Conclusions:**

TOA (35.4 ° ± 7.9 °) and L3 (26.8 ± 11.4 mm) could be used as a reference for ideal tibial tunnel placement in transtibial anatomic PCL reconstruction, so as to prevent recurrent PCL laxity and ensure good graft healing. However, further clinical validation is needed.

## Background

The transtibial tunnel technique has been commonly used for posterior cruciate ligament (PCL) reconstruction, in which the graft is pulled through and fixed in the tibial tunnel. Although the subjective knee scores were improved [1], clinical outcomes were not as predictable as that in anterior cruciate ligament (ACL) reconstruction [2–4]. Previous studies have demonstrated that the recurrent posterior laxity is one of the most common residual problems in PCL-reconstructed knees, which is related to thinning and elongation of the graft [1, 5, 6]. Inevitably, the graft will produce an acute bend around the proximal posterior tibia in transtibial tunnel technique [6, 7]. The acute bend is termed as “killer turn”. The more acute the “killer turn” for the graft emerging into the joint from a tibial tunnel, the higher risk of abrasion against the anterior ‘lip’ of the internal tibial tunnel aperture, thus leading to the enlargement of the tunnel inlet and attenuation of the graft [8].

Furthermore, previous studies had shown that the fixation strength of interference screw was significantly related to local thickness of cortical bone [9, 10]. And they recommended that fixation site should be placed away from the joint line. Accordingly, numerous surgeons tend to increase the tibial tunnel angle to achieve a smaller “killer turn” effect and stronger graft fixation. Whereas, several investigators had found in animal studies that the graft healing in cancellous-filled femoral tunnel was superior to that in marrow-dominated tibial tunnel [11–13]. They concluded that the healing of tendon-to-bone was highly correlated with peri-graft bone mass and connectivity, especially the cancellous bone architecture at the graft site. Because of the tibial marrow cavity, the peripheral cancellous bone mass of PCL graft would decrease with the increases in tibial tunnel angle. Thus leading to an adverse impact on graft healing. Therefore, there should be a compromise between reducing the “killer turn” angulation, increasing fixation strength and decreasing quality of bony tunnel. We speculated that the optimal position of the graft within the tibial tunnel was the proximal vertex of the tibial marrow cavity, which not only improve the “killer turn” angulation and fixation strength but also produce the satisfactory healing of tendon-to-bone. The theoretical optimal angle (TOA) is formed between the tibial tunnel through the proximal vertex of the tibial marrow cavity and tibial plateau.

The purpose of this study was to evaluate TOA of the tibial tunnel in transtibial anatomic PCL reconstruction through computed tomography (CT) measurements on the tibial anatomic characteristics.

## Methods

### Samples selection

After approved by the regional ethics committee of our institution, we retrospectively reviewed 963 CT images of the knee between January 2015 and August 2017 in our institution. Indications for CT were different from the aim of this study. Inclusion criteria: (1). patients accepted ultrahigh resolution CT examination of the knee; (2). the age range of patients is 18 years to 60 years; (3). scanning direction of CT was paralleled to anterior tibial crest, the selected sagittal section contained PCL tibial attachment and highest vertex of the tibial marrow cavity. Exclusion criteria: patients with displaced fractures involving knee, congenital skeletal dysplasia, previous knee surgery, inflammation or tuberculosis of bone and joint, tumor around the knee joint, and any knee abnormalities caused by disease.

### Computed tomography imaging

All included patients accepted standard clinical knee CT performed on a 64-multi-detector-row CT (SOMATOM Sensation, Siemens AG, Wittelsbacherplatz 2, Muenchen, Germany*)*. Scanning parameters included a gantry rotation speed of 1.00 s/rotation, 0.3 mm collimation width × 12 detectors, a CT pitch factor of 0.90 and a field of view of 25–30 cm. CT dose index (CTDI) volume was 20.9 mGy. Each patient was fixed in a supine position with the knee extended naturally.

### Measurements of the tibial anatomic characteristics

The measurements of the tibial anatomic characteristics were performed with the ST-PACS CDMedical software Vision 3.1 (Crealife, Beijing, China) by two independent, blinded observers. All CT images were evaluated by observer 1, and observer 2 measured 100 cases randomly selected from all specimens and blinded to results from observer 1. After 1 month, observer 1 measured 50 cases again which were randomly selected from all images. In this way, the intra- and inter-observer reliability were determined.

The measurements were taken on sagittal section that provided the most inclusive and wide PCL tibial attachment, including the thickness of cancellous bone (L1), the theoretical optimal angle of the tibial tunnel relative to plateau (TOA, producing the minimal “killer turn” effect on the premise of the satisfactory tunnel bone quality), L2 - the distance from anterior tunnel aperture to anterior end of tibial plateau, L3 - the distance from anterior tunnel aperture to tibial tuberosity (lowest edge of patellar ligament attachment). Surgeons could easily locate the tibial tunnel position during transtibial PCL reconstruction by measuring L2 and L3.

**Fig. 1:**
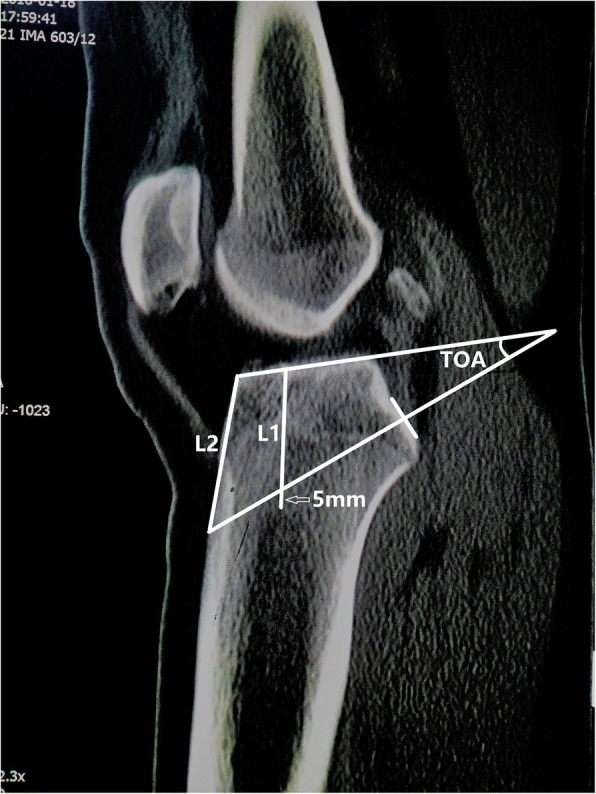
CT sagittal section: L1 was measured from the proximal vertex of the tibial marrow cavity to the tibial plateau along the anatomic axis of the marrow cavity; TOA was measured between the tibial plateau and the extension cord that connecting the center of PCL insertion site with a point 5 mm superior from the marrow cavity vertex (hollow white arrows); L2 was measured from from anterior tunnel aperture to anterior end of tibial plateau

**Fig. 2:**
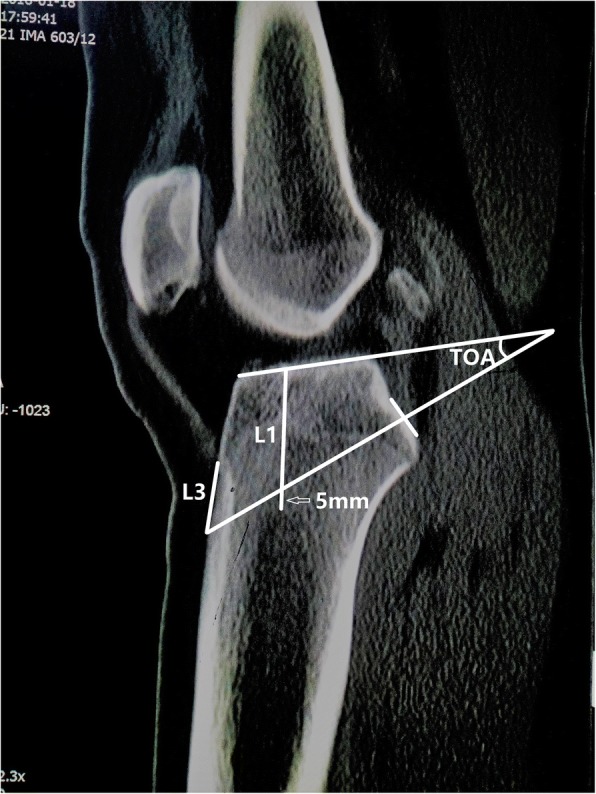
CT sagittal section: L3 was measured from anterior tunnel aperture to tibial tuberosity (lowest edge of patellar ligament attachment)

L1 was measured from the proximal vertex of the tibial marrow cavity to tibial plateau along the anatomic axis of marrow cavity, the proximal vertex was observed on sagittal section (Fig. 1). Subsequently, TOA was measured between tibial plateau and the extension cord that connecting the center of PCL insertion site with a point 5 mm superior from the marrow cavity vertex (Fig. 1).As described by Lee et al. [14], we chose the point 5 mm superior from the marrow cavity vertex because a 10-mm-diameter tibial tunnel was usually used in PCL reconstruction. L2 was measured from anterior end of tibial plateau to anterior tunnel aperture (Fig. 1). L3 was measured from the lowest edge of patellar ligament attachment site on tibia to anterior tunnel aperture (Fig. 2).

### Statistical analysis

All statistical analysis were performed using SPSS software (version 22.0, Inc., Chicago, IL, USA). The measurement results were shown as arithmetic mean ± standard deviation, to check up the data of all groups with normal test. All data were analyzed for the cohort as a whole, including age, height, and sex cohorts. One-way analysis of variance (ANOVA) was used to assess parameters among age cohorts and height cohorts, while independent t test between males and females. *P <* 0.05 was considered statistically significant. The intraclass correlation coefficients (ICCs) were used to analyze the calculation of intra- and inter-observer reliability. The ICCs ranges from 0.00 (no agreement) to 1.00 (perfect agreement).

## Results

In total, 408 knees were included in the present study (based on inclusion and exclusion criteria). There were 230 left knees and 178 right knees. The number was slightly higher in male patients (225) than that in female patients (183). The average age of patients was 38.3 ± 14.1 years (18–60 years in males, 18–60 years in females).

Based on sex, Table 1 showed the tibial anatomic parameters. The average value of TOA and L3 were 35.4 ± 7.9 ° and 26.8 ± 11.4 mm, respectively. There were no significant differences between sex groups (*P >* 0.05). The value of L1 and L2 were significant higher in males than that in females (L1, *P =* 0.002*;* L2, *P =* 0.046).

**Table 1:** Descriptive statistics of the proximal tibial parameters between males and females

Parameter	Mean Standard ± Deviation			P
	Total (*n* = 408)	Male (*n* = 225)	Female (*n* = 183)	
L1 (mm)	40.1 ± 7.6	41.1 ± 7.0	38.8 ± 8.0	.002
TOA (°)	35.4 ± 7.9	35.1 ± 7.2	35.8 ± 8.6	.352
L2 (mm)	47.0 ± 11.1	48.0 ± 10.8	45.8 ± 11.3	.046
L3 (mm)	26.8 ± 11.4	27.2 ± 10.9	27.3 ± 12.0	.463

*L1* the thickness of cancellous bone, *TOA* the theoretical optimal angle, *L2* the distance from anterior tunnel aperture to anterior end of tibial plateau, *L3* the distance from anterior tunnel aperture to the tibial tuberosity (lowest edge of patellar ligament attachment)

Regarding age, the relative results were shown in Table 2. We stratified the cases into three groups: the young (18–30 years old, *n* = 153), the middle-aged (31–45 years old, *n* = 104), the elderly (46–60 years old, *n* = 151). In total, the value of L1, TOA, L2 and L3 were significant higher in the middle-aged than the young (*P* = 0.02, *P* = 0.001, *P* = 0.038, *P* = 0.032, respectively). No significant differences were found in the value of measurements between the elderly and other age groups (*P > 0.05*).

**Table 2:** Anatomic parameters of the proximal tibia among three age groups

Age group (years)		Mean Standard ± Deviation			
		L1 (mm)	TOA (°)	L2 (mm)	L3 (mm)
Total	The Young 18–30 (n = 153)	39.2 ± 6.5	34.0 ± 6.7	45.6 ± 9.6	25.4 ± 10.6
	The Middle-aged 31–45 (*n* = 104)	41.6 ± 7.2*	37.3 ± 7.3*	48.9 ± 10.6*	28.7 ± 9.9*
	The Elderly 46–60 (*n* = 151)	39.9 ± 8.6	35.5 ± 9.1	47.2 ± 12.6	26.9 ± 12.9

Compared to the young, *P < 0.05. *L1* the thickness of cancellous bone, *TOA* the theoretical optimal angle, *L2* the distance from anterior tunnel aperture to anterior end of tibial plateau, *L3* the distance from anterior tunnel aperture to the tibial tuberosity (lowest edge of patellar ligament attachment)

With regard to height, we stratified the cases into three groups: I- < 1.66 m (*n* = 161), II - 1.66 to 1.75 m (*n* = 207) and III - > 1.75 m (*n* = 40), as shown in Table 3. The mean value of L1 was significant lower in group I than that in group II and group III (I *v* II, *P =* 0.015, I *v* III, *P =* 0.026). Similarly, the average value of L2 was also significant lower in group I than that in group II and group III (I *v* II, *P =* 0.026, I *v* III, *P =* 0.006). But there were no differences between group II and group III (*P > 0.05*). Interestingly, the average value of TOA and L3 showed no significant differences among three height groups (*P > 0.05*).

**Table 3:** Descriptive statistics of the proximal tibial parameters among three height groups

Height group (m)		Mean Standard ± Deviation			
		L1 (mm)	TOA (°)	L2 (mm)	L3 (mm)
Total	I- < 1.66 m (*n* = 161)	38.6 ± 8.2	35.5 ± 8.9	45.2 ± 11.6	25.7 ± 12.2
	II - 1.66 to 1.75 m (n = 207)	40.8 ± 6.7*	35.3 ± 7.1	47.8 ± 10.4*	27.0 ± 10.2
	III - > 1.75 m (*n* = 40)	42.4 ± 7.9*	35.4 ± 7.5	50.5 ± 11.5*	29.9 ± 13.2

Compared to I, *P < 0.05. *L1* the thickness of cancellous bone, *TOA* the theoretical optimal angle, *L2* the distance from anterior tunnel aperture to anterior end of tibial plateau, *L3* the distance from anterior tunnel aperture to the tibial tuberosity (lowest edge of patellar ligament attachment)

Inter- and intra-observer reliability of measurements were analyzed by ICCs. Table 4 shows the values were range from 0.641 to 0.909, indicating good reliability.

**Table 4:** Inter- and intraobserver reliability agreements of the proximal tibial parameters based on ICCs^a^

Parameter	Interobserver	Intraobserver
L1 (mm)	0.909	0.903
TOA (°)	0.721	0.641
L2 (mm)	0.793	0.859
L3 (mm)	0.817	0.891

^a^ *ICCs* intraclass correlation coefficients, *L1* the thickness of cancellous bone, *TOA* the theoretical optimal angle, *L2* the distance from anterior tunnel aperture to anterior end of tibial plateau, *L3* the distance from anterior tunnel aperture to the tibial tuberosity (lowest edge of patellar ligament attachment)

## Discussion

Present study evaluated the theoretical optimal angle (TOA) of the tibial tunnel on CT sagittal plane in transtibial anatomic PCL reconstruction. And the corresponding distance of TOA from anterior tunnel aperture to anterior end of tibial plateau (L2) and tibial tuberosity (L3) were also measured.

Previous studies have shown that clinical outcomes of PCL reconstruction were not so desirable compared with ACL reconstruction [2–4]. Many researchers believed that the “killer turn” for the graft emerging into the joint from a tibial tunnel could result in graft abrasion and tunnel inlet enlargement, which were critical causes to recurrent laxity after transtibial PCL reconstruction [6–8]. Meanwhile, animal studies suggested that the graft fixation strength with interference screw within the tibial tunnel was closely related to local thickness of cortical bone [9, 10, 15]. Therefore, surgeons tend to increase the tunnel angle to relieve the “killer turn” effect and increase the fixation strength. However, studies on rabbit models had shown that the peri-graft cancellous bone mass significantly influenced the healing of tendon-to-bone [11, 12]. For human tibias, the farther away from the knee joint line, the less the cancellous bone mass in proximal half. To our knowledge, this study firstly proposed the theoretical optimal angle of the tibial tunnel in transtibial anatomic PCL reconstruction that taking the “killer turn” effect, the graft fixation strength with interference screw and the healing of tendon-to-bone into account.

This study demonstrated that the average value of TOA, L2 and L3 were 35.4 ± 7.9 °, 47.0 ± 11.1 mm and 26.8 ± 11.4 mm, respectively. In other words, the graft will be located in marrow-dominated tunnel rather than cancellous-filled tunnel if a value of tunnel is higher than the corresponding limit. Thus, we speculated that there would be an adverse impact on the healing of tendon to bone according to the previous rabbit studies though they did not research tunnel angle [11, 12]. There is value in defining the optimal distances from anterior end of tibial plateau (L2) and tibial tuberosity (L3) to anterior tunnel aperture, but L2 is hard to clinically use due to difficulty in accuracy of palpation of tibial plateau. Actually, L3 could be positioned effortlessly on most people. Interestingly, L2 was influenced by sex, age and height of a different degree in the present study, while TOA and L3 were only influenced by age. Thus, TOA and L3 were relative constant references. X-ray measurement would be more accurate than direct vision or palpation to determine the value of TOA and L3 during PCL reconstruction. As a result, theoretical TOA (calculated by choice of ideal tunnel placement on sagittal CT images on this study) for transtibial anatomic PCL reconstruction was 35.4 ± 7.9 °. There was no effect of sex and height on TOA but it might be influenced by age.

### Limitations

A few limitations were present in our imageology study: (1) This study was conducted through CT images measurements, there were no definite CT data threshold to distinguish the cancellous bone and marrow cavity. Nevertheless, the deviation could be relieved by ultrahigh resolution CT of the knee in the study and our ICCs analysis also proved the good reliability of results. (2) The measurements were carried out in a single sagittal CT section without anatomic study, small deviation may exist. (3) We did not calculate a sample size. When we performed height subgroup analysis, the sample size of group III (> 1.75 m) was small. (4) The choice of an ideal tunnel placement is a theoretical study by imageology, there is no clinical validation presented. However, our results should be paid more attention by surgeons. In future, the authentic optimal position of the tibial tunnel should be comprehensively evaluated involving postoperative follow up, histological analysis, biomechanical study, etc.

## Conclusions

In summary, through the computed tomography (CT) measurements on the tibial anatomic characteristics, we found that the theoretical optimal angle of the tibial tunnel relative to tibial plateau and the corresponding distance from anterior tunnel aperture to the tibial tuberosity respectively were 35.4 ± 7.9 ° and 26.8 ± 11.4 mm in transtibial anatomic PCL reconstruction. However, further clinical validation is needed.

## Abbreviations

ACL: Anterior cruciate ligament
CT: Computed tomography
ICCs: Intraclass correlation coefficients
L1: The thickness of cancellous bone
L2: The distance from anterior tunnel aperture to anterior end of tibial plateau
L3: The distance from anterior tunnel aperture to tibial tuberosity (lowest edge of patellar ligament attachment)
PCL: Posterior cruciate ligament
TOA: The theoretical optimal angle of the tibial tunnel relative to tibial plateau
